# Supplementary material for: Cytoplasmic ribosomes on mitochondria alter the local membrane environment for protein import
Source: bioRxiv. 2024 Jul 19:2024.07.17.604013. Preprint. [Version 1] doi: 10.1101/2024.07.17.604013 (PMC11275913; doi:10.1101/2024.07.17.604013)
Supplement: 2 [file NIHPP2024.07.17.604013v1-supplement-2.pdf]

bioRxiv preprint doi: https://doi.org/10.1101/2024.07.17.604013; this version posted July 19, 2024. The copyright holder for this preprint (which was not certified by peer review) is the author/funder, who has granted bioRxiv a license to display the preprint in perpetuity. It is made available under aCC-BY 4.0 International license.

SUPPLEMENTARY FIGURES

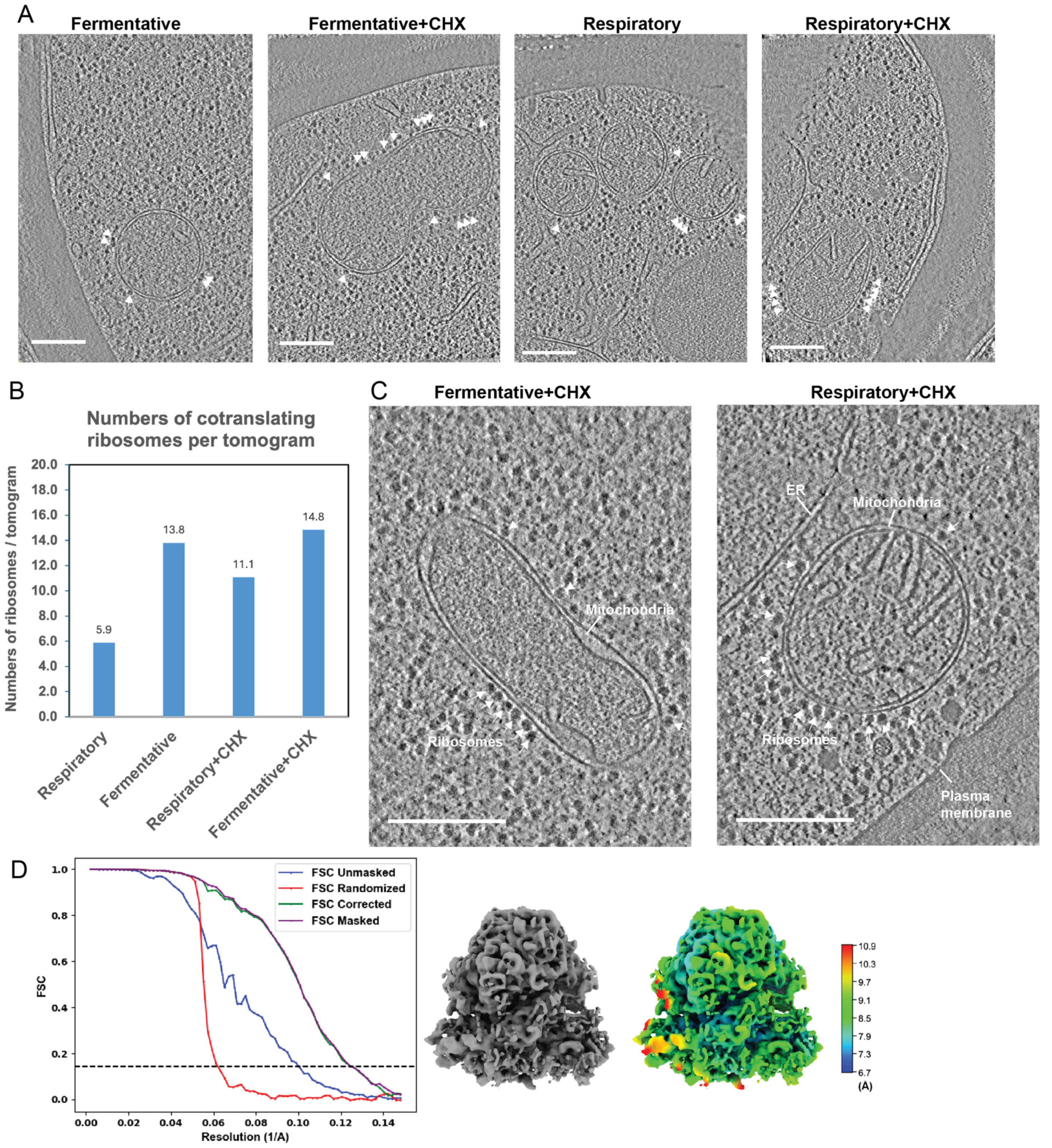

Supplementary Figure 1. Representative tomograms of cryo-focused ion beam (cryo-FIB) tomograms milled *S. cerevisiae* cell lamellae with visible mitochondria-associated cytoplasmic ribosomes.

A. Representative X-Y slices of reconstructed tomograms collected at pixel size 2.638 Å from cryo-FIB milled *S. cerevisiae* yeast cells grown in different growth conditions (i.e., fermentative and respiratory) and treatment

bioRxiv preprint doi: https://doi.org/10.1101/2024.07.17.604013; this version posted July 19, 2024. The copyright holder for this preprint (which was not certified by peer review) is the author/funder, who has granted bioRxiv a license to display the preprint in perpetuity. It is made available under aCC-BY 4.0 International license.

conditions (i.e., vehicle and cycloheximide, CHX). Cytoplasmic ribosomes in close proximity to the OMM are highlighted by white arrowheads. Scale bars = 250nm

B. Quantification of the number of ribosomes positioned with the exit tunnel facing the OMM in CHX-treated or vehicle-treated cells grown in respiratory versus fermentative conditions.

C. Representative X-Y slices of reconstructed tomograms collected at pixel size 1.6626 Å from cryo-FIB milled *S. cerevisiae* grown in fermentative and respiratory conditions and treated with CHX (100 μg/mL) displaying subcellular features such as mitochondria, ribosomes, the endoplasmic reticulum, and the plasma membrane. Scale bars = 250 nm

D. Fourier shell correlation plot (left) of the 80S cytoplasmic ribosome reconstruction is shown with resolution reported at 0.143 FSC and the reconstructed subtomogram average (middle) shown to the right of the curves. The 80S cytoplasmic ribosome was resolved to 8 Å from 35,784 ribosome particles with the color map (right) shows the local resolution.

bioRxiv preprint doi: https://doi.org/10.1101/2024.07.17.604013; this version posted July 19, 2024. The copyright holder for this preprint (which was not certified by peer review) is the author/funder, who has granted bioRxiv a license to display the preprint in perpetuity. It is made available under aCC-BY 4.0 International license.

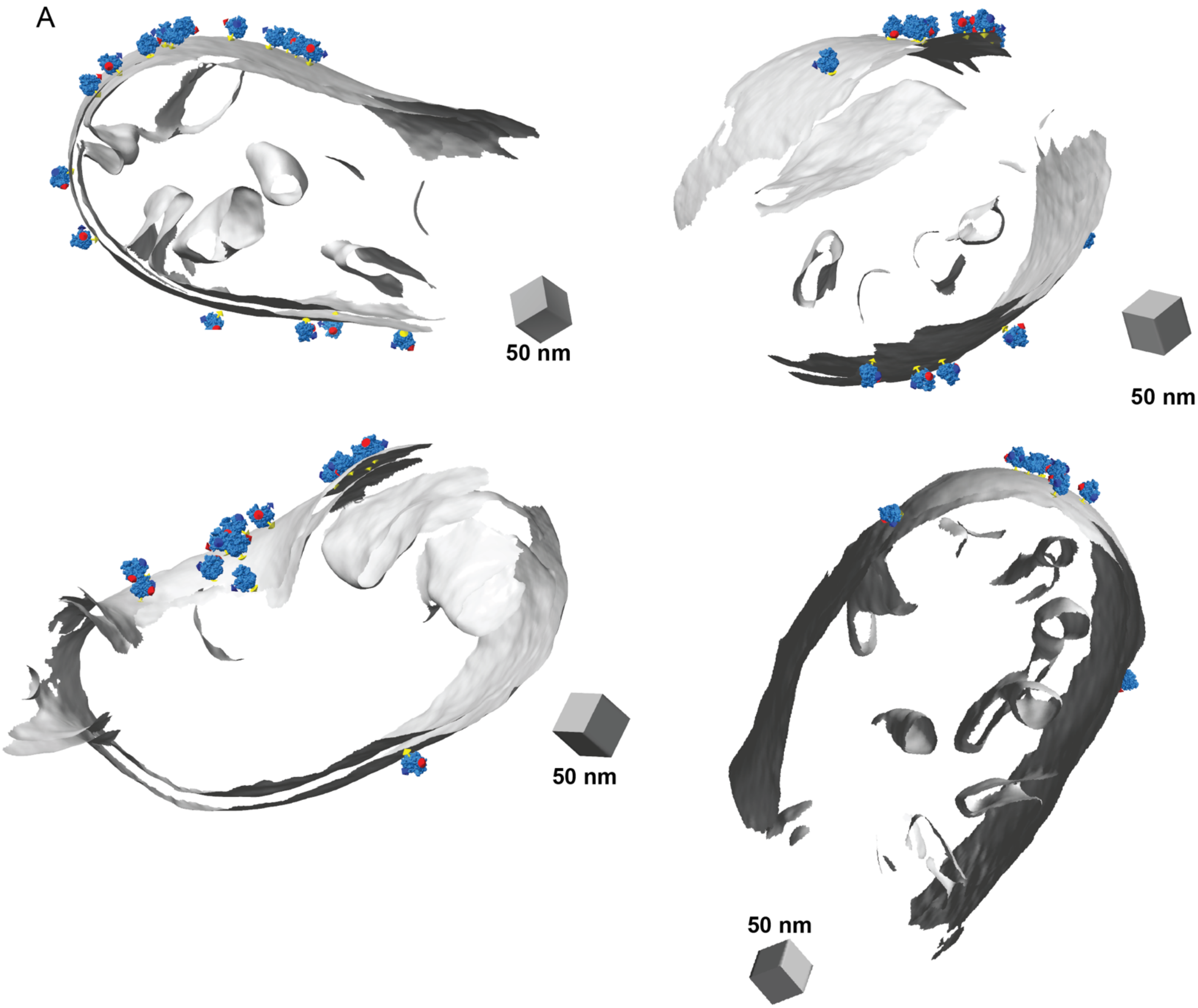

Supplementary Figure 2. Distance filtering identifies a subset of cytoplasmic ribosomes optimally positioned for protein import into the OMM.

A. A subset of representative models of ribosomes positioned with their exit tunnels optimally positioned for protein import on the OMM surface.

bioRxiv preprint doi: https://doi.org/10.1101/2024.07.17.604013; this version posted July 19, 2024. The copyright holder for this preprint (which was not certified by peer review) is the author/funder, who has granted bioRxiv a license to display the preprint in perpetuity. It is made available under aCC-BY 4.0 International license.

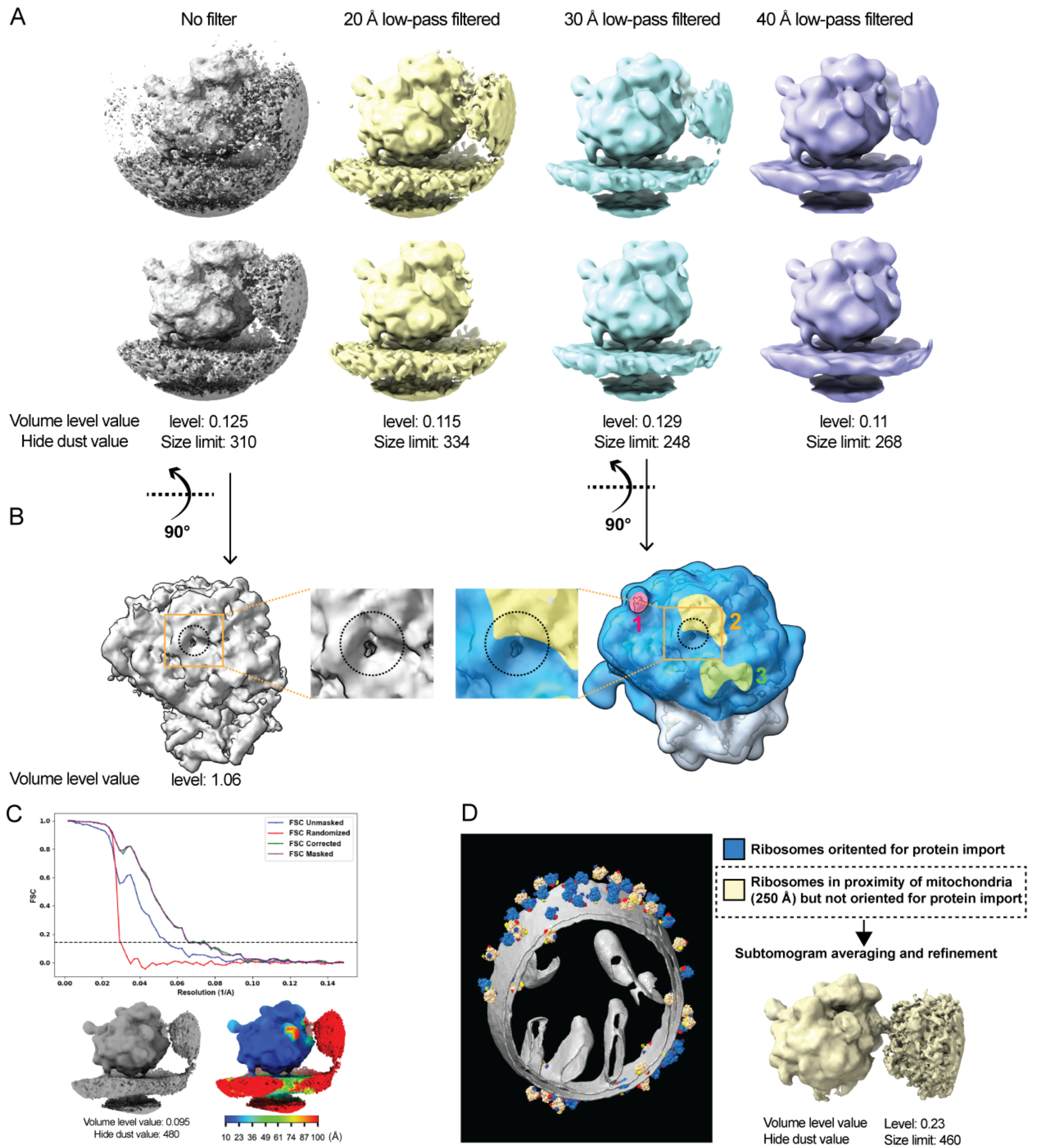

Supplementary Figure 3. Three-dimensional subtomogram average of a cytoplasmic ribosome positioned for protein import on the outer mitochondrial membrane (OMM).

A. Subtomogram average of a cytoplasmic ribosome positioned for protein import on the OMM displayed with varying levels of low-pass filter, isosurface volume threshold, and hide dust values in ChimeraX. The densities surrounding the ribosome on the cytoplasmic side, as observed in other studies (Brandt, Carlson et al. 2010, Pfeffer, Brandt et al. 2012, Gemmer, Chaillet et al. 2023), likely correspond to neighboring ribosomes.

bioRxiv preprint doi: https://doi.org/10.1101/2024.07.17.604013; this version posted July 19, 2024. The copyright holder for this preprint (which was not certified by peer review) is the author/funder, who has granted bioRxiv a license to display the preprint in perpetuity. It is made available under aCC-BY 4.0 International license.

B. The peptide exit tunnel is visible in the subtomogram average (gray density) at lower isosurface volume thresholds, as indicated by the dashed black line. This was used to mark its position relative to the connecting densities visible in the subtomogram average at higher isosurface volume threshold values (colored density).

C. Fourier shell correlation plot of the OMM-associated 80S cytoplasmic ribosome reconstruction is shown with resolution reported at 0.143 FSC and the reconstructed subtomogram average (bottom left) shown to the right of the curves. The 80S cytoplasmic ribosome was resolved to 19 Å from 1,076 ribosome particles with the color map shows the local resolution (bottom right).

D. Representative models of ribosomes positioned within 250 Å of the OMM surface with their exit tunnels facing away from the OMM. 3D refinement of these particles results in 3D reconstructions of a ribosome that does not contain any distinguishable connecting densities between the 80S ribosome and the OMM, suggesting that these connections are specific to 80S ribosomes optimally positioned for protein import.

bioRxiv preprint doi: https://doi.org/10.1101/2024.07.17.604013; this version posted July 19, 2024. The copyright holder for this preprint (which was not certified by peer review) is the author/funder, who has granted bioRxiv a license to display the preprint in perpetuity. It is made available under aCC-BY 4.0 International license.

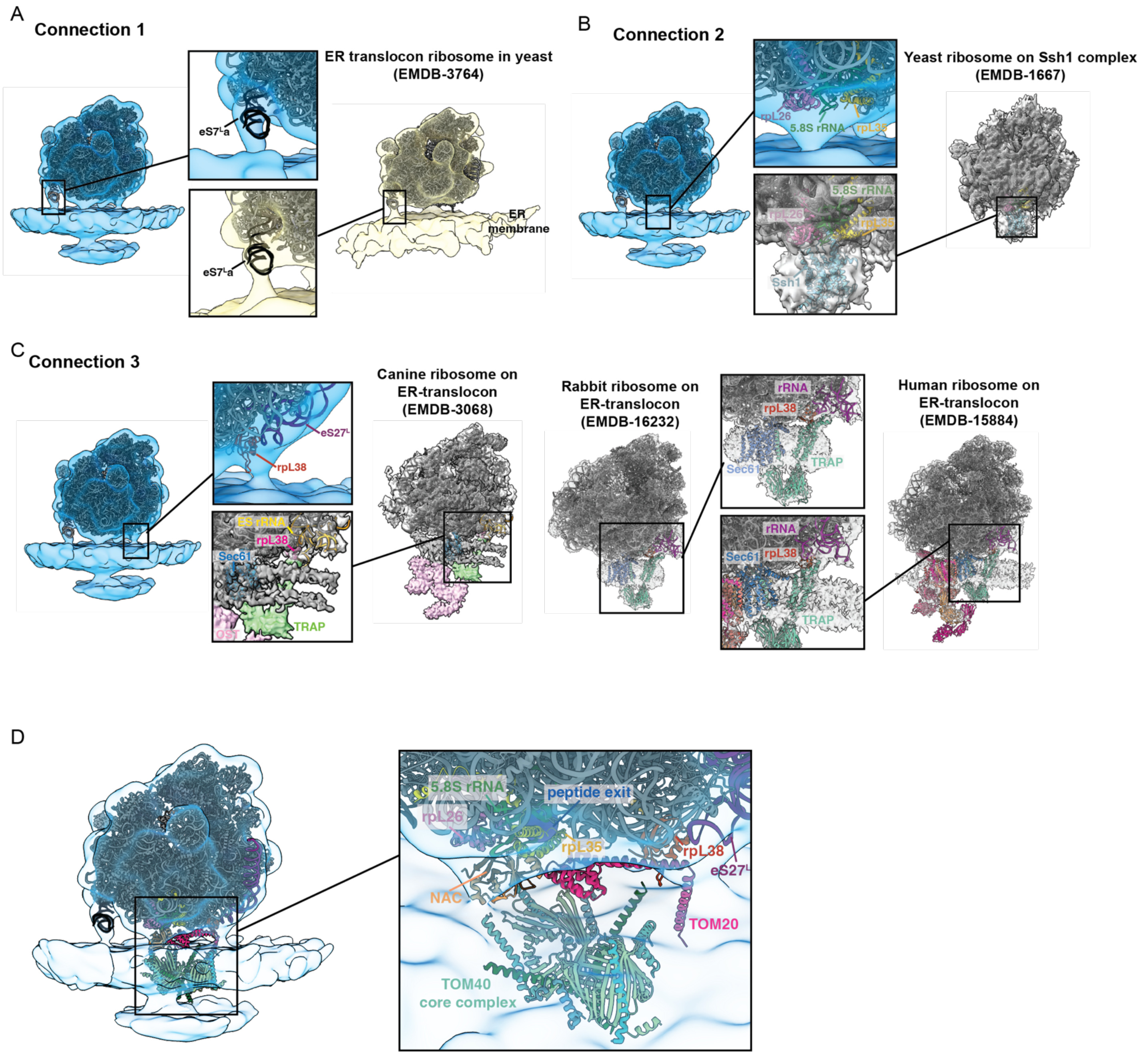

Supplementary Figure 4. Similarities between the organization of the mitochondrial-associated ribosome and ER-associated ribosome structures, and the hypothesized model for the architecture of mitochondrial co-translational import.

A. Density corresponding to the connection labeled #1 in the subtomogram average of mitochondrial-associated ribosomes correlates well with the density corresponding to the expansion segment of eS7La of the 25S rRNA in the large 60S subunit present in the ER-associated ribosome maps from *S. cerevisiae* (EMD-3764).

B. Density corresponding to the connection labeled #2 correlates well with the density corresponding to the region where ribosome interacts with the import channel, Ssh1, in the ER-associated ribosome maps from *S. cerevisiae* (EMD-1667).

bioRxiv preprint doi: https://doi.org/10.1101/2024.07.17.604013; this version posted July 19, 2024. The copyright holder for this preprint (which was not certified by peer review) is the author/funder, who has granted bioRxiv a license to display the preprint in perpetuity. It is made available under aCC-BY 4.0 International license.

C. Density corresponding to the connection labeled #3 correlates well with the density corresponding to the rRNA expansion segment, rpL28, and the translocon-associated protein complex (TRAP) in the ER-associated ribosome maps from human (EMD-15884), rabbit (EMD-16232), and canine (EMD-3068).

D. The hypothesized model of the arrangement of cytoplasmic ribosomes and components associated with the mitochondrial membrane generated by docking atomic models of these components from previous work (Jomaa, Gamerdinger et al. 2022, Gamerdinger, Jia et al. 2023, Ornelas, Bausewein et al. 2023) into our subtomogram average.

bioRxiv preprint doi: https://doi.org/10.1101/2024.07.17.604013; this version posted July 19, 2024. The copyright holder for this preprint (which was not certified by peer review) is the author/funder, who has granted bioRxiv a license to display the preprint in perpetuity. It is made available under aCC-BY 4.0 International license.

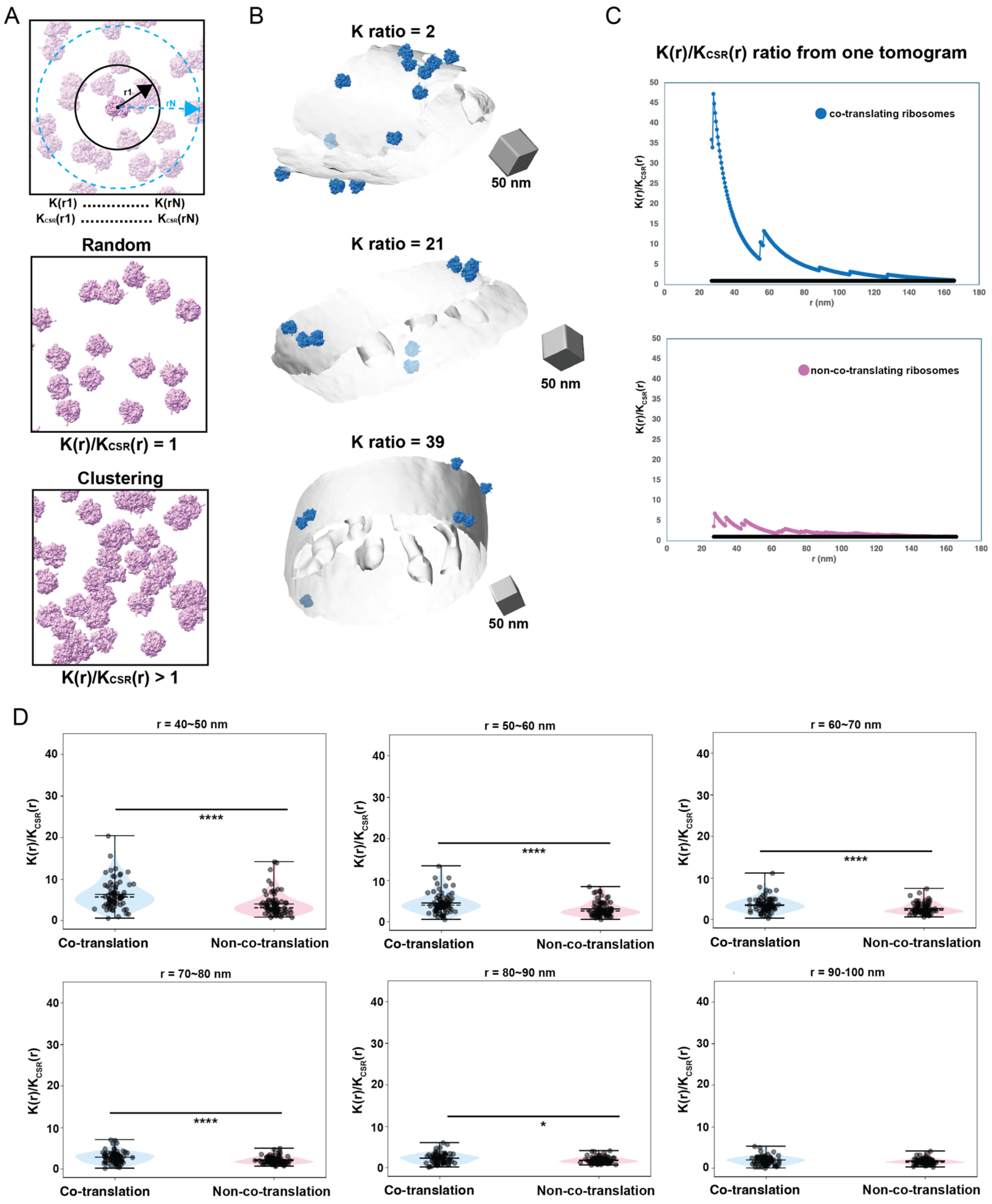

Supplementary Figure 5. Cytoplasmic ribosomes primed for protein import cluster on the mitochondrial membrane.

A. Visual representation of the Ripley's K function analysis defining the degree of ribosome clustering within a given radius (r) of the indicated ribosome (top panel). The example of spatial organization patterns shows random

bioRxiv preprint doi: https://doi.org/10.1101/2024.07.17.604013; this version posted July 19, 2024. The copyright holder for this preprint (which was not certified by peer review) is the author/funder, who has granted bioRxiv a license to display the preprint in perpetuity. It is made available under aCC-BY 4.0 International license.

(middle panel) and clustering (bottom panel) distribution with their corresponding ratio from the analysis. Images are adapted from (Martin-Solana, Diaz-Lopez et al. 2024).

B. Representative models of ribosomes oriented for import and membranes from tomograms display different $K(r)/K_{CSR}(r)$ ratios.

C. Representative plots for the for the $K(r)/K_{CSR}(r)$ ratio for mitochondrial-associated cytoplasmic ribosomes oriented for protein import within a range of radius (r) values of 27-166 nm. The black line equals to 1.

D. Quantification of the maximum value of $K(r)/K_{CSR}(r)$ for each tomogram at the indicated radius intervals for each ribosome class. P values from Mann-Whitney U test are indicated. *P < 0.05; **P < 0.01; ***P < 0.005; ****P < 0.001.

bioRxiv preprint doi: https://doi.org/10.1101/2024.07.17.604013; this version posted July 19, 2024. The copyright holder for this preprint (which was not certified by peer review) is the author/funder, who has granted bioRxiv a license to display the preprint in perpetuity. It is made available under aCC-BY 4.0 International license.

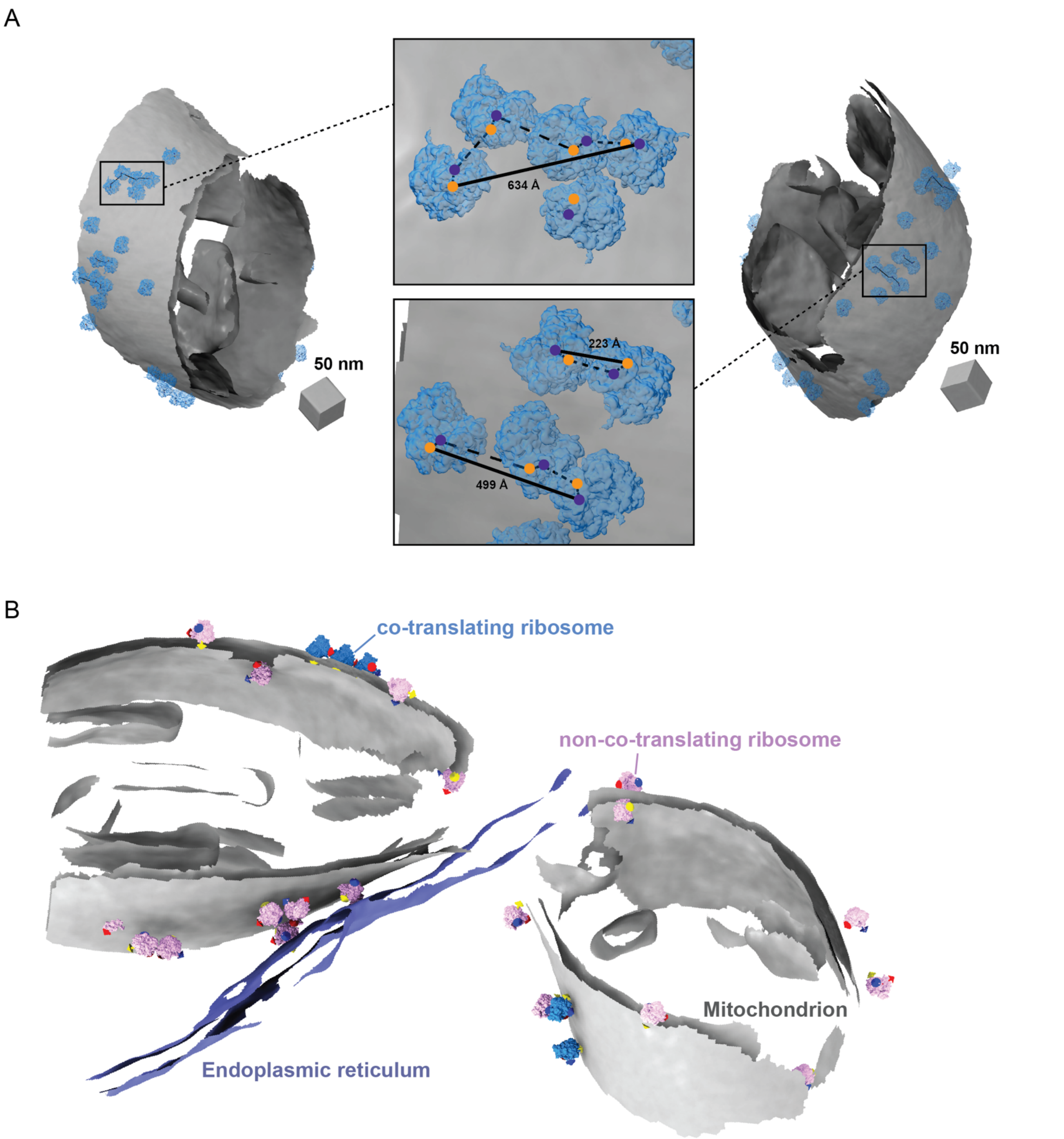

Supplementary Figure 6. Cytoplasmic ribosomes primed for protein import cluster on the mitochondrial membrane.

A. Representative membrane surface reconstructions of mitochondria (gray) with ribosomes oriented for import relative to the OMM (blue). Insets show zoomed-in boxed regions of the ribosome models with circle overlays demarking the location of the 3’ mRNA entry (blue), the 5’ mRNA exit sites (orange), the possible pathways of interconnecting mRNA (dashed black line), and the calculated end-to-end distance from 5’ to 3’ of each interconnected mRNA (solid black line).

bioRxiv preprint doi: https://doi.org/10.1101/2024.07.17.604013; this version posted July 19, 2024. The copyright holder for this preprint (which was not certified by peer review) is the author/funder, who has granted bioRxiv a license to display the preprint in perpetuity. It is made available under aCC-BY 4.0 International license.

B. Membrane surface reconstruction of mitochondria (gray) and endoplasmic reticulum (blue) membranes with corresponding models for co-translating (blue) and non-co-translating ribosomes (pink).

bioRxiv preprint doi: https://doi.org/10.1101/2024.07.17.604013; this version posted July 19, 2024. The copyright holder for this preprint (which was not certified by peer review) is the author/funder, who has granted bioRxiv a license to display the preprint in perpetuity. It is made available under aCC-BY 4.0 International license.

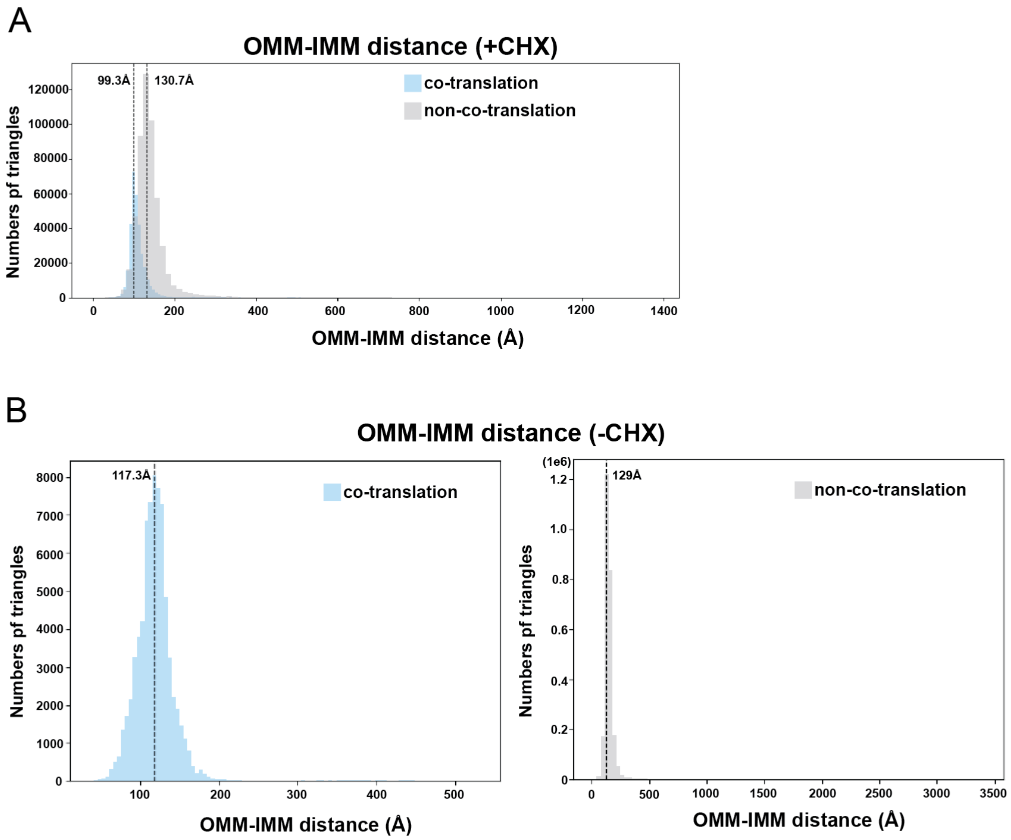

Supplementary Figure 7. Ribosome-associated protein import alters the local architecture of the outer and inner mitochondrial membranes.

A. Combined histogram of IMM-OMM distances of co-translation-associated and non-co-translation-associated patches in *S. cerevisiae* treated with CHX. Dashed vertical lines correspond to peak histogram values of pooled data.

B. Histograms of IMM-OMM distances of co-translation-associated and non-co-translation-associated patches in *S. cerevisiae* treated with vehicle (e.g., no CHX). Dashed vertical lines correspond to peak histogram values of pooled data.

Supplementary Movie 1. Three-dimensional subtomogram average of a cytoplasmic ribosome optimally positioned for protein import on the outer mitochondrial membrane (OMM).
